# Idiopathic Hyperprolactinemia: Clinical Presentation, Diagnosis, Management, and Clinical Outcome

**DOI:** 10.7759/cureus.98701

**Published:** 2025-12-08

**Authors:** Olabosipo Sholuade, Goodness Njoku, Suma Kaza, Reshma A Fatteh, Manoj Kollukkad, Charles Edwards

**Affiliations:** 1 Medical School, Avalon University School of Medicine, Willemstad, CUW; 2 Pathology, Avalon University School of Medicine, Willemstad, CUW; 3 Behavioral Health and Psychiatry, Avalon University School of Medicine, Willemstad, CUW; 4 Human Structure and Function, Avalon University School of Medicine, Willemstad, CUW; 5 Obstetrics and Gynecology, Avalon University School of Medicine, Willemstad, CUW

**Keywords:** bromocriptine therapy, dopamine agonists, hyperprolactinemia and mood disorders, hyperprolactinemia diagnosis, idiopathic hyperprolactinemia, pcos and hyperprolactinemia, pituitary imaging, prolactin dysregulation

## Abstract

Polycystic ovary syndrome (PCOS) is a multifactorial endocrine disorder often presenting with menstrual irregularities, hyperandrogenism, and metabolic dysfunction. Hyperprolactinemia, characterized by elevated prolactin levels, may share overlapping features with PCOS and complicate diagnosis. When these two conditions coexist, they present unique clinical and management challenges.

A 25-year-old woman with a history of PCOS presented with amenorrhea, progressive weight gain, and irregular menstrual cycles. Laboratory investigations revealed persistently elevated prolactin levels, while pituitary imaging ruled out structural abnormalities. The patient received lifestyle modification advice and dopamine agonist therapy, with continuous monitoring of hormonal, metabolic, and psychological parameters.

This case highlights the diagnostic complexity of idiopathic hyperprolactinemia occurring in the context of PCOS, and emphasizes the importance of a multidisciplinary approach to management. Further clinical observations are needed to understand the neuropsychiatric effects of prolactin dysregulation.

## Introduction

Hyperprolactinemia, defined as elevated serum prolactin levels, is an endocrine disorder that frequently presents with menstrual irregularities, galactorrhea, and infertility [1,2]. It is most commonly caused by pituitary adenomas (prolactinomas) but may also result from medications, hypothyroidism, or stress [2,3]. The diagnosis of hyperprolactinemia requires careful evaluation to determine potential etiologies and to exclude structural causes [1,2]. Importantly, elevated prolactin levels have been associated with mood disturbances, including anxiety and depressive symptoms, likely due to their influence on serotonin and dopamine pathways [4,5]. Several systematic reviews and meta-analyses support a relationship between hyperprolactinemia and neuropsychiatric manifestations. However, the strength of this association may vary and should be considered on a case-by-case basis [4,5].

This case report explores the diagnostic challenges and therapeutic approach in a patient presenting with idiopathic hyperprolactinemia while discussing the potential implications of prolactin dysregulation on mood disorders as a possible, but not definitive, clinical manifestation. Although mood disturbances are highlighted in current literature, the patient did not endorse any such symptoms during evaluation, underscoring the heterogeneity in clinical presentation.

## Case presentation

A 25-year-old female with a past medical history of polycystic ovary syndrome (PCOS) presented with a 10-month history of amenorrhea and progressive weight gain. She previously experienced irregular menstrual cycles occurring every 4-6 months until September 2011. Her medical history included an abnormal Pap smear (grade 3A), indicating minor dysplasia. Blood pressure readings were mildly elevated on two occasions (131/83 mmHg and 130/94 mmHg). Physical examination revealed no galactorrhea, significant hirsutism, or acne. Pelvic and abdominal examinations were unremarkable. She denied the use of medications known to elevate prolactin levels, including antipsychotics, antidepressants, opiates, and antiemetics, which further supported an idiopathic etiology.

Laboratory findings

The initial biochemical evaluation revealed elevated serum prolactin with features suggestive of a hyperandrogenic state. The detailed laboratory results are summarized in Table 1. Serial prolactin assessments demonstrated significant variability associated with treatment adherence, as summarized in Table 2.

**Table 1:** Baseline hormonal and biochemical profile LH: Luteinizing hormone; FSH: Follicle-stimulating hormone; TSH: Thyroid-stimulating hormone; β-hCG: Human chorionic gonadotropin; PCOS: Polycystic ovary syndrome

Test	Result	Normal Reference Range	Interpretation
Prolactin	90.0 ng/mL	4.8–23.3 ng/mL (female)	Elevated; consistent with hyperprolactinemia
LH	5.5 IU/L	2.4–12.6 IU/L (follicular phase)	Within normal range
FSH	2.9 IU/L	3.5–12.5 IU/L (follicular phase)	Slightly low; consistent with PCOS pattern
Testosterone	118 ng/dL	15–70 ng/dL (female)	Elevated; supports hyperandrogenic state
Progesterone	30 ng/dL	<150 ng/dL (follicular phase)	Within expected range
Estradiol	76 pg/mL	30–120 pg/mL (follicular phase)	Normal
TSH	1.04 µIU/mL	0.4–4.0 µIU/mL	Normal thyroid function
β-hCG	<5 mIU/mL	<5 mIU/mL (non-pregnant)	Confirms non-pregnant status

**Table 2:** Prolactin levels at two timepoints

Date	Prolactin (ng/mL)	Notes
March 2010	97.1 ng/mL	Peak level due to non-compliance with bromocriptine
October 2010	9.4 ng/mL	Lowest level achieved with adherence to therapy

The patient was started on bromocriptine (Parlodel) 1.25 mg orally once daily. This dosing regimen was selected as the recommended starting dose to improve tolerability and gradually normalize prolactin levels.

## Discussion

The clinical complexity of idiopathic hyperprolactinemia presents significant diagnostic and management challenges, particularly in cases where structural causes such as pituitary adenomas must be ruled out. In this case, imaging of the pituitary region was conducted by the treating physician, excluding pituitary adenoma; however, the actual images were not printed or made available for inclusion in this manuscript due to financial and logistical limitations. The radiology report described a normal pituitary gland, midline pituitary stalk, and no evidence of microadenoma or macroadenoma, further strengthening the diagnosis of idiopathic hyperprolactinemia.

Hyperprolactinemia is often linked to secondary causes such as medications, hypothyroidism, and stress, but when these are excluded, it is classified as idiopathic. This case illustrates the diagnostic uncertainty in hyperprolactinemia, as no clear underlying etiology was identified. The patient’s prolactin levels fluctuated in response to treatment with bromocriptine, supporting a functional dysregulation rather than an autonomous prolactin-secreting adenoma [1-3].

The role of prolactin in mood regulation has been increasingly recognized. Prolactin interacts with multiple neurotransmitter systems, including dopamine and serotonin, which play key roles in regulating mood, motivation, and emotional well-being. Excess prolactin can lead to decreased dopamine levels, contributing to depressive and anxiety symptoms. Several reviews and meta-analyses report that endocrine disorders, including hyperprolactinemia, frequently manifest with neuropsychiatric symptoms such as emotional lability, fatigue, and depressive episodes [4,5]. Patients with untreated hyperprolactinemia have been reported to exhibit higher rates of depressive symptoms, emotional instability, and reduced cognitive function, which may improve with appropriate management. However, it is important to note that the patient in this case denied experiencing depressive or anxiety symptoms, and her psychological status remained stable throughout follow-up.

Further complicating the relationship between hyperprolactinemia and mood disorders is its potential impact on sleep patterns and fatigue levels. Elevated prolactin has been associated with disrupted sleep architecture, increased daytime sleepiness, and impaired overall energy levels, all of which may exacerbate depressive symptoms [4,5]. These observations highlight the importance of incorporating mental health assessments and interventions into the comprehensive management strategy for hyperprolactinemia.

Diagnostic challenges and the role of imaging

Although the imaging studies could not be physically reproduced in this manuscript, they were completed as part of the patient’s clinical workup and did not reveal any pituitary lesion or structural abnormality. These findings, in combination with prolactin levels below the 100 ng/mL threshold and a favorable response to dopamine agonist therapy, support the diagnosis of idiopathic hyperprolactinemia. Guidelines from the Endocrine Society recommend imaging, preferably MRI, for all patients with unexplained hyperprolactinemia to assess for prolactinomas or other sellar pathology [1,2]. In resource-limited settings where full radiographic documentation may not be possible, clinical findings, hormonal profiles, and therapeutic response can guide decision-making; however, formal imaging assessment remains the gold standard. The absence of mass-effect symptoms, such as headaches or visual field deficits, further reduced the likelihood of an underlying macroadenoma.

Therapeutic approach and long-term management

Dopamine agonists, such as bromocriptine and cabergoline, remain the cornerstone of hyperprolactinemia treatment, effectively reducing prolactin levels and restoring ovulatory function [2,3]. In this case, periodic normalization of prolactin levels was achieved with bromocriptine therapy; however, non-compliance resulted in fluctuations of prolactin. Cabergoline has been shown to be more effective and better tolerated for long-term therapy than bromocriptine [3].

Long-term follow-up is essential in patients with hyperprolactinemia to monitor for recurrence and assess for late-onset complications, such as osteoporosis and psychiatric disorders. Chronic hyperprolactinemia has been associated with decreased bone mineral density, emphasizing the need for routine bone health evaluation [6]. Similarly, given the evidence linking hyperprolactinemia to mood disturbances, periodic mental health assessments should be integrated into follow-up care [4,5]. In this patient, continued monitoring was emphasized despite the absence of overt psychiatric symptoms.

Implications for future research

Further studies are needed to explore the neuropsychiatric and metabolic implications of hyperprolactinemia, particularly in idiopathic cases. Research focusing on neurotransmitter changes could provide insights into the link between prolactin dysregulation and mood disorders. Additionally, prolactin may contribute to insulin resistance, independent of conditions such as PCOS, suggesting a more direct metabolic role [7]. Understanding these mechanisms could guide more targeted and effective treatment strategies for affected patients.

## Conclusions

Idiopathic hyperprolactinemia presents significant diagnostic and therapeutic challenges, particularly when it coexists with conditions such as PCOS and when documentation is limited by resource constraints. This case underscores the importance of thorough hormonal evaluation, confirmation of structural integrity through imaging, and consideration of the broader neuropsychiatric impact of elevated prolactin levels. While mood disturbances are not universally present, they should be actively monitored as part of comprehensive care. A multidisciplinary approach, including endocrinology, gynecology, and mental health support, is essential to effectively manage the physiological and potential psychological dimensions of this condition. Further research is warranted to better understand the neuroendocrine mechanisms underlying hyperprolactinemia-related mood disturbances and to refine treatment strategies accordingly.
